# Doxycycline reduces MMP-2 activity and inhibits invasion of 12Z epithelial endometriotic cells as well as MMP-2 and -9 activity in primary endometriotic stromal cells in vitro

**DOI:** 10.1186/s12958-019-0481-z

**Published:** 2019-04-13

**Authors:** Eleftherios P. Samartzis, Daniel Fink, Manuel Stucki, Patrick Imesch

**Affiliations:** Division of Gynecology, University Hospital Zurich, Frauenklinikstrasse 10, CH-8091 Zürich, Switzerland

**Keywords:** Endometriosis, Cell culture, Extracellular matrix, Progesterone, Female reproductive tract

## Abstract

**Background:**

Matrix metalloproteinases (MMPs), especially the gelatinases MMP-2 and MMP-9, play a crucial role in the pathogenesis of endometriosis by enabling invasion. Doxycycline is a well-tolerated antibiotic and a potent MMP-inhibitor in subantimicrobial doses.

**Methods:**

Gelatin zymography and activity assays were used to detect latent and active MMP-2 and -9 in cell culture supernatants of immortalized epithelial (12Z) and two isolates of primary endometriotic stromal cells treated with doxycycline. The invasiveness of 12Z endometriotic cells treated with doxycycline was assessed in matrigel-coated invasion chambers. The effect on latent and active MMP-2 expression of the combination of progesterone and doxycycline was tested in 12Z.

**Results:**

Doxycycline significantly reduced the MMP-2 activity and pro-MMP-2 expression in 12Z and the MMP-2 and -9 activity as well as expression of pro-MMP-2 and -9 in primary endometriotic stromal cells. The percentage of 12Z cells invading through a matrigel-coated membrane was reduced to 65 and 22% of the control after treatment with doxycycline at doses of 1 μg/ml and 10 μg/ml, respectively. Furthermore, a combination of progesterone and doxycycline showed an additive effect in low doses on the reduction of MMP-2 activity and pro-MMP2 expression in 12Z endometriotic cells.

**Conclusions:**

In conclusion, the MMP-inhibiting features of subantimicrobial-dose doxycycline may be further evaluated as a well-tolerable additional therapeutic approach, e.g. in combination with progestins such as dienogest, in patients with infiltrative endometriosis with insufficient response to current medical treatment options.

## Background

One of the most important pathogenic characteristics of the proliferation of endometriosis, especially in the deep-infiltrating form, is the invasion of endometriotic cells through the basilar membrane of the peritoneal mesothelium into the extracellular matrix [1]. Although surgical resection of endometriotic lesions is the standard therapeutic approach in symptomatic endometriosis, recurrence of the disease and its symptoms after surgery is frequent and often requires repeated surgeries [2]. Treatment strategies of endometriosis associated with pain are the combination of surgical removal of endometriotic lesions followed by a medical prophylaxis for recurrence. At present, no clinically available medical compound for the treatment of endometriosis is cytoreductive. Therefore, the suppression of new implants rather than the elimination of existing lesions should be the goal of any postoperative pharmacological treatment [3]. Although medical treatment with GnRH analogues and more recently with dienogest (a synthetic progestin) have proven to be efficacious to a certain extent, there are frequent cases where these therapies are not sufficient to control endometriosis and to prevent a recurrence of the disease [4]. Consequently, combinatory treatments with other compounds may be a promising option to increase the efficacy of the already available therapies used against endometriosis and non-hormonal drugs may be an interesting alternative for patients wishing a non-hormonal medical prevention of a recurrence of endometriosis which is still yet not available.

Matrix metalloproteinases (MMPs), especially members of the group of gelatinases (MMP-2 and MMP-9), play a crucial role in the development of endometriosis, since MMP-9 has been shown to be increased in eutopic and ectopic endometrial tissue from women with endometriosis and higher levels of MMP-2, − 9, and − 14 mRNA have been found in endometriotic cells when compared to normal endometrium [5–7]. Furthermore, the concentration of MMP-2 has been shown to be significantly elevated in the serum and peritoneal fluid of women with endometriosis in comparison to healthy women [8]. Consequently MMPs produced by endometriotic cells may degrade the extracellular matrix leading to vascularization and growth of endometriotic lesions and invasion into the peritoneal layer [9]. The pathogenic role of MMP-9 has also been demonstrated in endometrial epithelial cells of patients with endometriosis [10]. Specific inhibitors that exhibit a similar action to the endogenous antagonists, the tissue inhibitors of metalloproteinases (TIMPs), such as ONO-4817, have shown promising results in animal models in the treatment of e.g. endometriosis uteri interna, also known as adenomyosis [11]. However, excessive TIMP levels may also be associated with adverse events leading to reproductive problems [12] and inhibitors similar to endogenous TIMP may therefore not be suitable for the treatment of endometriosis [13]. However, the roles and interactions of different MMPs in endometriosis are complex and not yet fully understood [14].

Doxycycline, a well-known antibiotic substance of the family of the tetracyclines is a well-tolerated drug that interestingly also possesses strong MMP inhibitory activity that is already observed at a subantimicrobial dosage level [15, 16]. This effect was first observed in periodontitis research, and clinical studies are investigating its use as an MMP inhibitor in dermatology, cardiovascular medicine, ophthalmology and dentistry [16, 17]. The MMP-inhibiting effect of subantimicrobial-dose doxycycline relies on a direct inhibition of the active form of MMPs, which is achieved by the binding of calcium and zinc ions as well as by a direct inhibition of the activation of latent pro-MMPs [15].

The aim of this study was to investigate if doxycycline acts as an inhibitor of MMP expression and activity in endometriotic cells in vitro. Therefore, an immortalized epithelial endometriotic cell line (12Z), which is widely used in endometriosis research, and since there was no access to an immortalized stromal cell line (e.g. 22B) for the stromal compartment, primary stromal endometriotic cells isolated from endometriomas were studied in the experiments. Furthermore, using 12Z in matrigel coated membranes we studied if doxycycline inhibits the invasion of endometriotic cells. Moreover, we aimed to investigate whether a combinatory treatment with low-dose doxycycline and progesterone increases the MMP-inhibiting effect of these drugs in 12Z endometriotic cells.

## Methods

### Cell culture

This study was conducted on an immortalized epithelial endometriotic cell line (12Z) and on two primary stromal cells freshly isolated from endometriotic tissue as described below. Mycoplasma testing of the cultured cells was carried out on a regular basis during the study and was repeatedly negative. The primary cell study was approved by the institutional review board and individual informed consent was obtained by the participants. Only early passage numbers were used for the experiments (in 12Z: passage 2–5 of the initially received cell line, in primary stromal cells: passage 2–3 of freshly isolated cells).

### Immortalized epithelial endometriotic cell line 12Z

The 12Z endometriotic cell line was kindly provided by Professor Anna Starzinski-Powitz (Department of Biology, University of Frankfurt, Frankfurt am Main, Germany). The cell line was generated through the in situ electroporation of primary peritoneal epithelial endometriotic cells with SV-40 T-antigen. The characteristics of this cell line have been previously described [18]. 12Z cells were cultured in Dulbecco’s Modified Eagle Medium (DMEM, 21980; Invitrogen, Basel, Switzerland) containing 10% fetal bovine serum (Gibco, LifeTechnologies, Waltham, MA, USA) and 1% antibiotic-antimycotic (containing penicillin, streptomycin and amphotericin B, Gibco) at 37 °C in an atmosphere with 5% CO_2_ and 95% humidity. Experiments were performed with a cell confluency of 50–70%.

### Primary cell cultures

Primary cells were isolated from histologically confirmed endometriotic tissue of two different patients by the following procedure: Endometriotic tissue samples from endometriomas were collected freshly in the operating room and transferred directly to the cell culture lab for further processing. The diagnosis of endometriosis was confirmed histologically by a pathologist. The freshly collected endometriotic tissue was separated from any connective tissue and then minced in fine slices and incubated for 90 min in a 10 mg/ml collagenase solution at 37 °C (Collagenase C2674, Sigma-Aldrich, Saint Louis, MO, USA). After this digestion procedure, the suspension was passed through a 100-μm mesh filter to separate the cells from any remaining connective tissue. The filtrate was then collected and passed through a second 40-μm mesh filter. The flow through, consisting of the stromal endometriotic cells, was transferred to a plate and cultured under standard culturing conditions in Iscoves modified medium (IMEM, Gibco) supplemented with 10% fetal bovine serum and 1% antibiotic-antimycotic (containing penicillin, streptomycin and amphotericin B, Gibco) in an incubator at 37 °C in an atmosphere of 5% CO_2_ and 95% humidity. For the drug treatment experiments cell confluency was 80% at the time point of treatment. Primary epithelial endometriotic cells were not kept in culture since low cell quantity and proliferation rate did not allow an adequate MMP quantification in these cells. As part of a parallel experiment and confirmation of stromal origin of the cells we performed three-dimensional (3D) cell cultures with the primary stromal cells (not shown). The cells aggregated to 3D-cell-conglomerates and we then produced histological slides from the microtissues, which were stained immunohistochemically for pan-cytokeratin, vimentin, and mib-1. Vimentin showed a general strong positive staining and pan-cytokeratin stained ubiquitously negative. Mib-1 stained positive in a few cells. In addition, some brown pigment was found which most likely corresponds to blood residues.

### Drug treatment

For the drug treatment experiments, 12Z or primary stromal endometriotic cells were seeded in 6-well plates at a density of 3 × 10^5^ cells per well for 12Z and 6 × 10^5^ cells per well for primary stromal cells. The following day, the medium was replaced by serum-free medium after washing the cells once with phosphate-buffered saline. After 24 h the medium was replaced with 2 ml of serum-free medium containing drugs in the indicated doses. Serum-free medium was used to avoid foreign protease activity in the serum as has been done by others [19] and no signs of reduced cell viability were found as described below. Doxycycline (doxycycline hyclate, Sigma-Aldrich) was purchased and stock solutions were diluted in sterile water to a concentration of 1 mg/ml and kept in the dark at − 20 °C. Treatment doses were chosen according to cell culture doses for human cell lines reported in the literature [20]. Progesterone (Sigma-Aldrich) was stored at − 20 °C as a stock solution in DMSO at a concentration of 100 mM. Drugs were freshly diluted from the stock for every treatment. Controls were treated with the vehicle substance in the same concentration.

### Cell viability

Cell viability after treatment with doxycycline was assessed in parallel to the drug treatment experiments described above with an automated cell-counting device (Countess®, Invitrogen, LifeTechnologies, Thermo Fisher Scientific Inc., Waltham, MA, USA) after trypan blue staining of the cells to distinguish viable from dead cells. After the 24 h doxycycline treatment, the supernatants were collected and adherent cells were trypsinized and resuspended in the respective supernatants. Ten microliters of the cell suspension were mixed 1:1 with trypan blue staining and filled into the counting chamber. The number and fraction of viable cells was determined for each plate and calculated as percentage of the number of viable cells in the control plate.

### Gelatin zymography

Gelatin zymography was used to detect gelatinases in the supernatants of treated cells as has been done by others [19]. Briefly, supernatants of treated cells (a total of 25 μl per well containing *v*/v 1:1 of supernatant and Laemmli buffer) were loaded on a gelatin (type A, G-8150, Sigma-Aldrich) containing SDS polyacrylamide gels (acrylamide concentration of 8%) followed by electrophoresis for 6 h. SDS and Triton X-100 were then washed out of the gels to allow a renaturation of the MMP proteins. This was followed by incubation for 24 h (if not specified as prolonged incubation of 36 h) in a zinc- and calcium-chloride-containing buffer at 37 °C, allowing gelatin degradation by gelatinases. Gels were stained with Coomassie blue for 2 h and then destained for 45 to 90 min until bands representing gelatin degradation by the gelatinases became visible. Reconstituted lyophilized human pro-MMP-2 and pro-MMP-9 were used as positive controls (contained in Biotrak MMP-2 and MMP-9 activity assays kits, GE Healthcare). The gelatin zymograms were quantified with the Image-J software (imagej.nih.gov/ij).

### MMP activity assay

Levels of the active form of MMP-2 and MMP-9 were measured by an activity assay (Biotrak MMP-2 activity assay and Biotrak MMP-9 activity assay, GE Healthcare) according to the provided manufacturer’s protocol. Briefly, supernatants of treated cells were added to the anti-MMP-2 (respectively anti-MMP-9) precoated microplate wells and incubated overnight at 4 °C in accordance with the manufacturer’s instructions. The following day, the plates were rinsed 4 times with the provided washing buffer and then incubated with the assay buffer in addition to the substrate and detection reagent. The plates were analyzed immediately after the addition of the detection reagent (a dection enzyme that is activated by captured active MMP-2 (respectively MMP-9) through a single proteolytic event, which can be measured using a specific chromogenic peptide substrate) for the baseline measurement and again after 3-6 h for signal recording in a spectrophotometer (Epoch Microplate Spectrophotometer, BioTek, Winooski, VT, USA). Standard curves were obtained for each activity assay and were similar to the standard curve provided by the manufacturer.

### Matrigel invasion assay

The matrigel invasion assay was carried out in 24-well matrigel invasion chambers (BD BioCoat BD Matrigel Invasion Chamber, Franklin Lakes, New Jersey 07417–1880). In brief, 12Z endometriotic cells were seeded in invasion chambers (2.5 × 10^4^ cells per 24-well invasion chamber) in serum-free medium containing the indicated doses of doxycycline. Serum-containing medium (DMEM containing 10% fetal bovine serum) was added to the lower chamber as a chemoattractant and the chambers were placed in an incubator at 37 °C and 5% CO_2_ for 22 h. The cells that did not migrate through the matrigel membrane were subsequently removed with a cotton swab and the cells at the bottom of the membrane were fixated, stained with crystal violet and counted manually by bright field microscopy on an inverted microscope (Leica DMI6000B).

### Statistical analysis

Statistics were performed with GraphPad Prism version 7.02 (GraphPad Software Inc., La Jolla, CA, USA) using ANOVA analysis followed by a Fisher LSD test. Results are represented as mean ± standard error of mean (SEM) in all experiments with three or more independent replicates (mean ± standard deviation (SD) in experiments with two independent replicates) and significance (only calculated in experiments with at least three replicates) was assumed if *p* ≤ 0.05.

## Results

### Doxycycline reduces MMP-2 activity and expression of pro-MMP-2 in immortalized endometriotic cells (12Z)

**Fig. 1:**
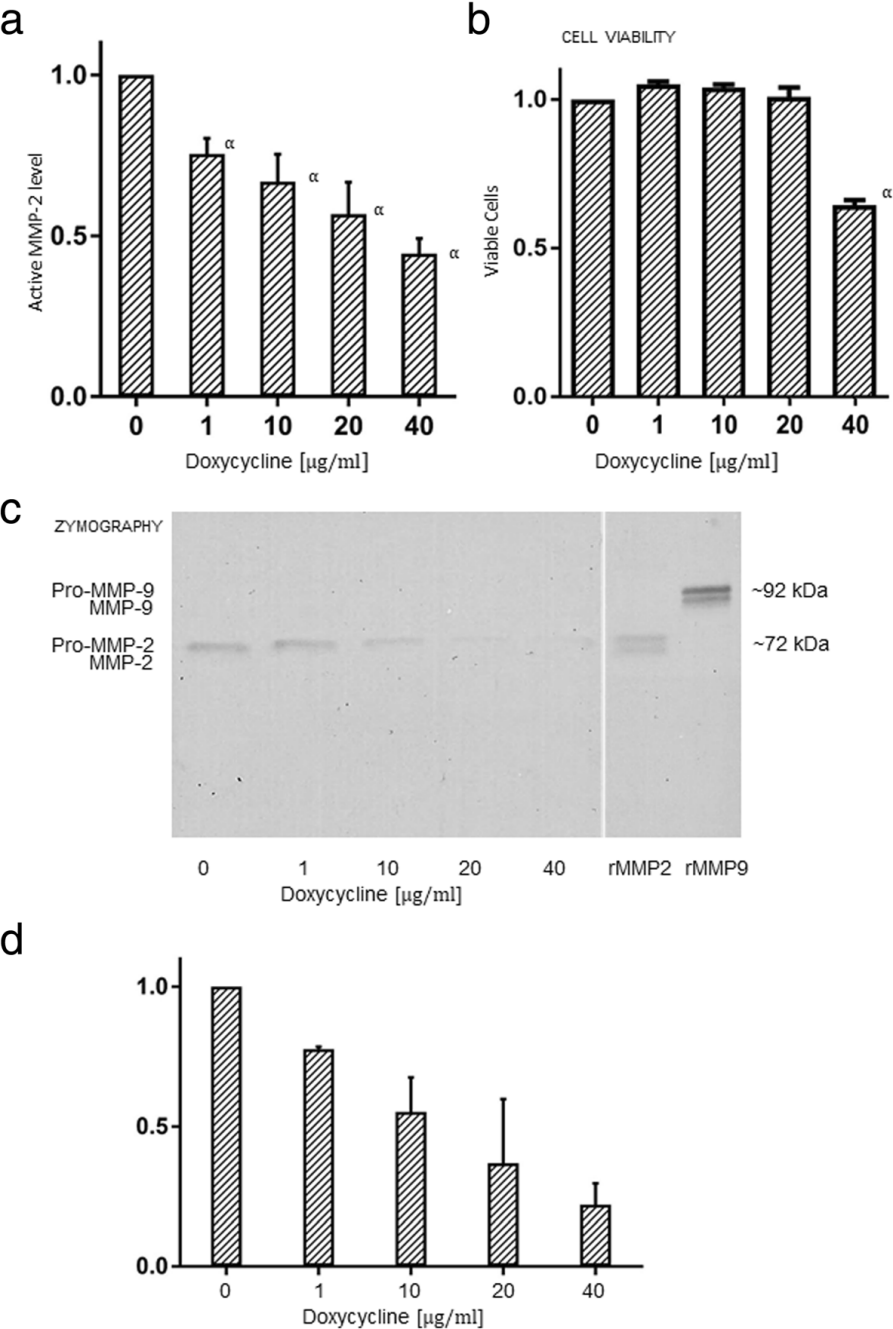
Doxycycline reduces active MMP-2 levels in 12Z endometriotic cells. **a** Determination of the active form of MMP-2 in doxycycline-treated 12Z endometriosis cells in an activity assay (Biotrak MMP-2 activity assay) which specifically binds MMP-2 in antibody-coated microwells followed by repeated washing steps. The treatment with doxycycline reduced the active MMP-2 levels in a dose-dependent manner. The mean values of three independent treatments are represented with the standard error of mean (SEM) and significance labeled as “α” if *p* ≤ 0.05. **b** Cell viability was assessed with trypan blue to exclude that reduced MMP-2 levels were due to increased cell death rates. As is shown in the figure, concentrations of doxycycline up to 20 μg/ml did not lead to reduced cell viability. Representation of the mean and SEM, significant differences (i.e. if p ≤ 0.05) are labeled with an “α” next to the bar. **c** Representative illustration of a zymography gel (inverse picture) for detection of latent and active gelatinase levels ((pro-)MMP-2 and -9). The analysis was performed in culture supernatants of immortalized 12Z endometriotic cells treated with respective doses of doxycycline in serum-free medium for 24 h. Samples were loaded on an electrophoresis gel containing gelatin, followed by an incubation for 24 h in a buffer medium containing zinc and calcium chloride at 37 °C. The molecular size of the detected bands corresponds to pro MMP2 and the bands showed decreasing expression with increasing doses of doxycycline. Note the second band below pro MMP2 corresponding to active MMP-2 which is not visible in the samples probably due to the very low amount compared to the latent form. (Pro-)MMP-9 was not detectable in 12Z by gelatin zymography. rMMP-2 and -9: reconstituted lyophilized human pro-MMP-2 and pro-MMP-9. **d** Quantification of pro-MMP-2 activity from two independent drug treatments. Representation of the mean and standard deviation (SD)

Doxycycline treatment of 12Z epithelial endometriotic cells reduced the detected activity of MMP-2 in a dose-dependent manner in the MMP-2 activity assay (Fig. 1a). To exclude cell mortality as a reason for reduced MMP-2 secretion, we performed viability assays. We observed no significant cell mortality up to doses of 20 μg/ml doxycycline, whereas 40 μg/ml doxycycline led to a reduced number of viable cells (65% of the control, Fig. 1b). This means that the reduced levels of MMP-2 up to 20 μg/ml were not due to reduced cell numbers or increased mortality of endometriotic cells. Reduced expression of latent MMP-2 (pro-MMP-2) was found in a zymography assay upon treatment with doxycycline (Fig. 1c and d).

### Doxycycline reduces MMP-2 and MMP-9 activity and expression of pro-MMP-2 and pro-MMP-9 in primary endometriotic cells

**Fig. 2:**
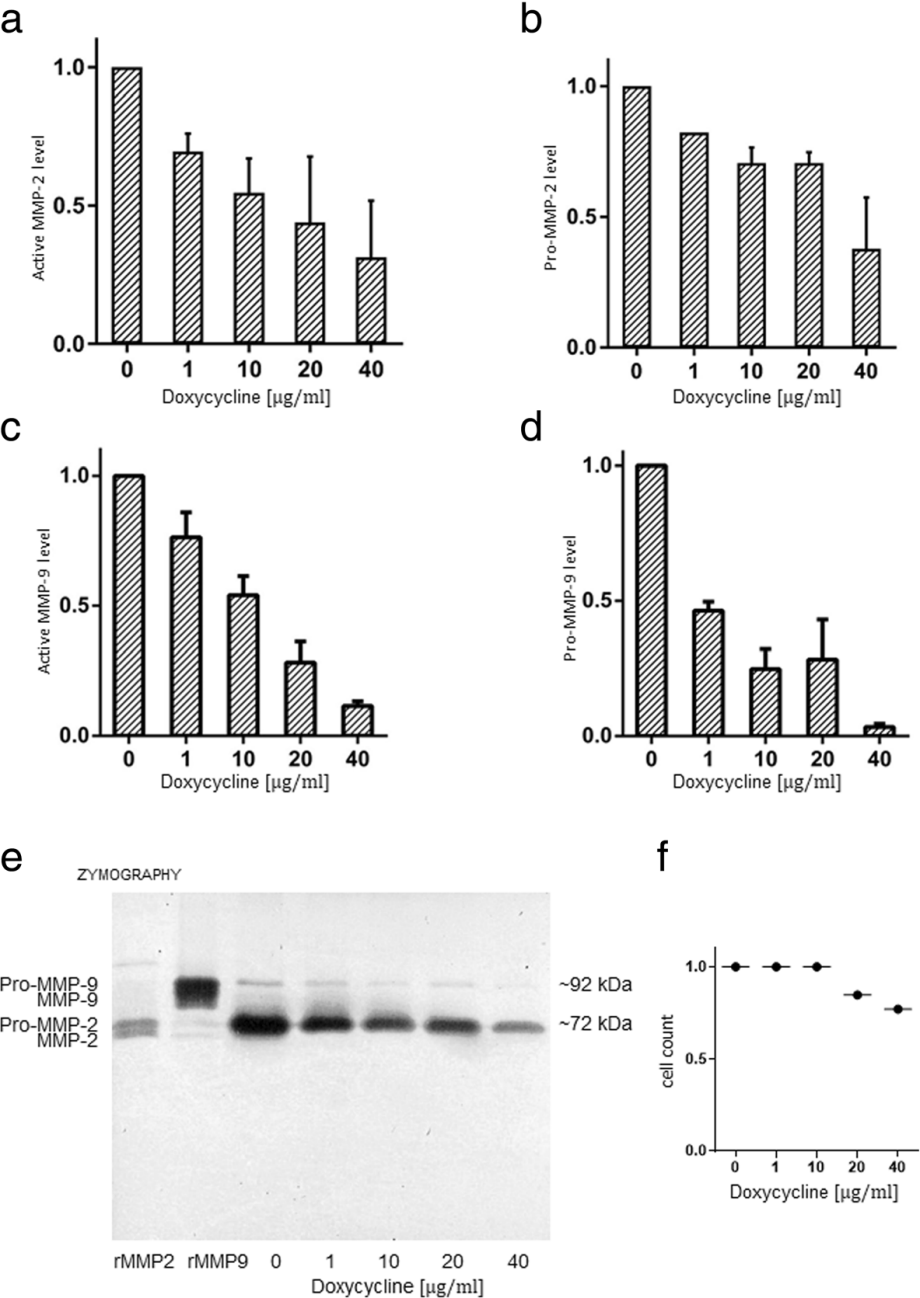
Doxycyline treatment reduces active MMP-2 and -9 levels in primary stromal endometriotic cells. **a** and **b** Dose-dependent decrease of latent and active MMP-2 levels following treatment with doxycycline as detected by zymography (**a**) and in an activity assay (**b**) in primary stromal endometriotic cells. In contrast to the immortalized epithelial endometriotic cell line 12Z, MMP-9 was also detectable in the supernatants of stromal endometriotic primary cell cultures. Similarly to MMP-2, treatment with doxycycline led to a dose-dependent decrease of active MMP-9 levels, examined by zymography (**c**) and in activity assays (**d**). Note that the activity assay specifically detects one MMP (− 2 or − 9) per assay by specific antibody binding and several washing steps (i.e. washing out of potential interfering inhibitors or activators) in the antibody-coated microwells, whereas zymography is performed by direct loading of the supernatants and therefore reflects the situation “as it is”. This is the reason that both different methods were performed and are represented here. It also explains that the graphs of the different methods are not identical. **e** Representative picture of a zymography gel loaded with the supernatant of primary cell cultures of endometriotic stromal cells (inverse picture). The bands corresponding to pro-MMP-2 and -9 are clearly visible. Note the second band below corresponding to the activated form of MMP-2 and -9 which is also very lightly visible in the untreated control. Doxycycline treatment led to a dose-dependent reduction of levels of pro-MMP-2 and -9. Representation of the mean values and SD of two independent experiments. **f** Primary stromal cells were automatically counted after treatment with doxycycline compared to the control. The number of adherent cells with normal microscopical shape was identical up to 10 μg/ml of doxycycline compared to the control, whereas reduced cell numbers were counted when treated with 20 μg/ml and 40 μg/ml, which microscopically correlated with more visible detached dead cells and cell debris

Primary endometriotic stromal cells were obtained from two different patients and extracted from endometriotic tissue issuing from deep-ovarian endometriomas as described above. Analysis of the presence of gelatinases by zymography revealed high levels of latent MMP-2 (pro-MMP-2) and moderate levels of latent MMP-9 (pro-MMP-9) in the supernatants of both primary stromal cell cultures (cell passage number: 2). Doxycycline treatment led to a dose-dependent reduction of MMP-2 and MMP-9 activity in the primary endometriotic cells (Fig. 2a and c) on cell passage number 3. A reduction of pro-MMP-2 and pro-MMP-9 levels in doxycycline-treated primary endometriotic stromal cells (cell passage number 3) was observed in zymography analyses (Fig. 2b, d and e). There were no microscopic signs of increased cell death and same cell counts in primary stromal endometriotic cells after treatment with doxycycline up to 10 μg/ml compared to the control, whereas some signs of increased cell death and reduced cell counts were observable in cells treated with 20 μg/ml and more pronounced with 40 μg/ml as shown in Fig. 2f.

### Doxycycline inhibits the invasion of 12Z through matrigel membranes

**Fig. 3:**
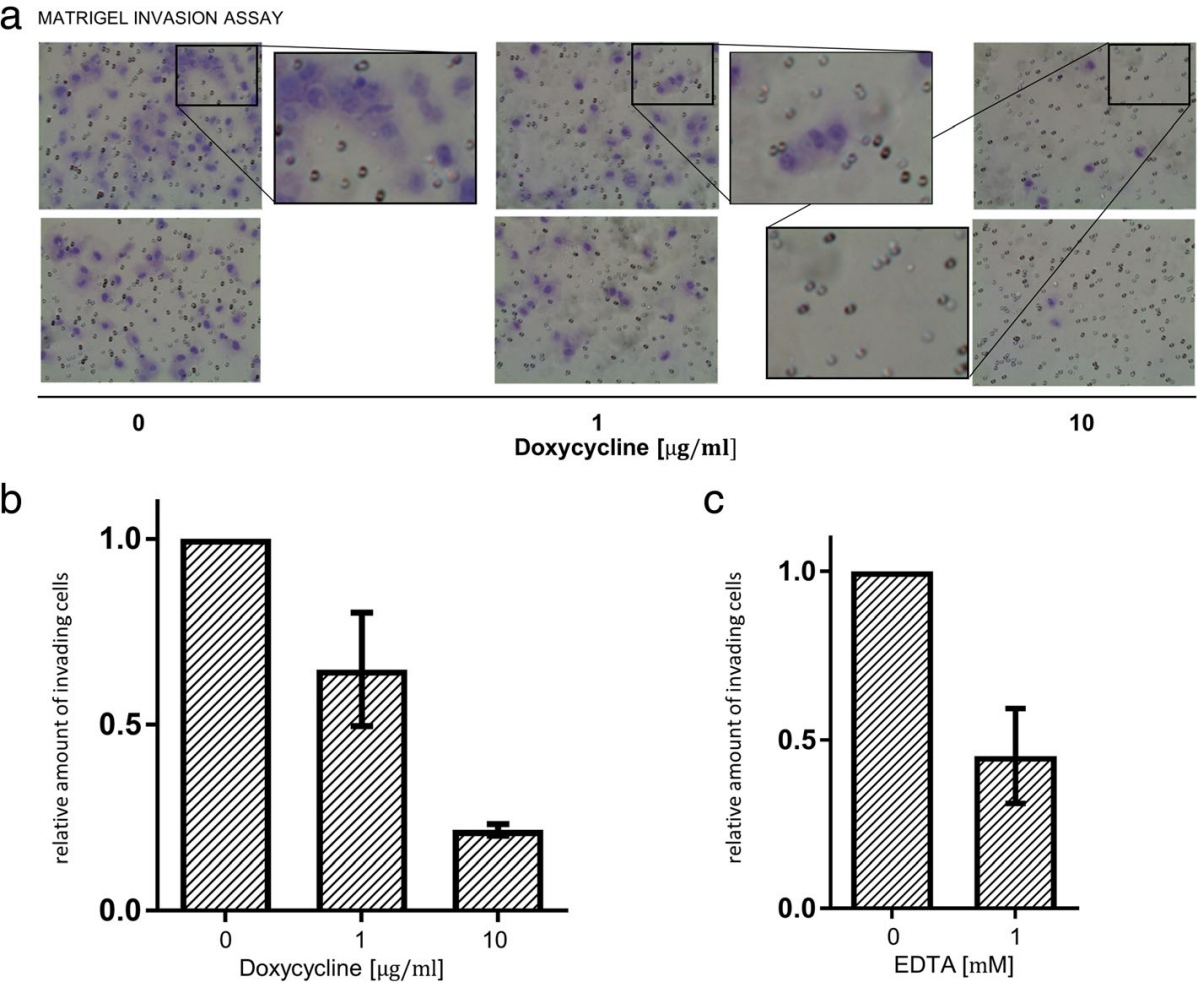
Doxycycline reduces the invasion of 12Z endometriotic cells in dose dependent manner. **a** Representative pictures of two matrigel invasion assays: 12Z endometriotic cells were seeded into matrigel-coated invasion chambers and treated with respective doses of doxycycline in serum-free medium. Serum-containing medium (DMEM containing 10% fetal bovine serum) was added to the lower chamber as a chemoattractant and the invasion chambers were incubated for 22 h at 37 °C and 5% CO_2_. Subsequently, the invaded cells were fixed, stained with crystal violet and counted using a microscope. The violet-stained endometriotic cells are clearly distinguishable from the much smaller, unstained pores of the matrigel membrane. **b** Relative number of invaded cells compared to the untreated control. Result of two independent experiments performed in duplicate each. **c** Control treatment with EDTA which inhibits the activity of gelatinases by binding of zinc. The results are represented as mean ± SD

Using matrigel-coated invasion chambers, we observed a reduced invasion of doxycycline treated 12Z endometriotic cells compared with the control. Treatment with doxycycline at a concentration of 1 μg/ml reduced the relative number of invading cells to 65% and 10 μg/ml reduced the invasive fraction to 22% of the control (Fig. 3a and b). EDTA 1 mM was used as a positive control (Fig. 3c).

### Combination treatment of progesterone and doxycycline synergistically reduces the MMP-2 activity and expression of pro-MMP-2 in 12Z

**Fig. 4:**
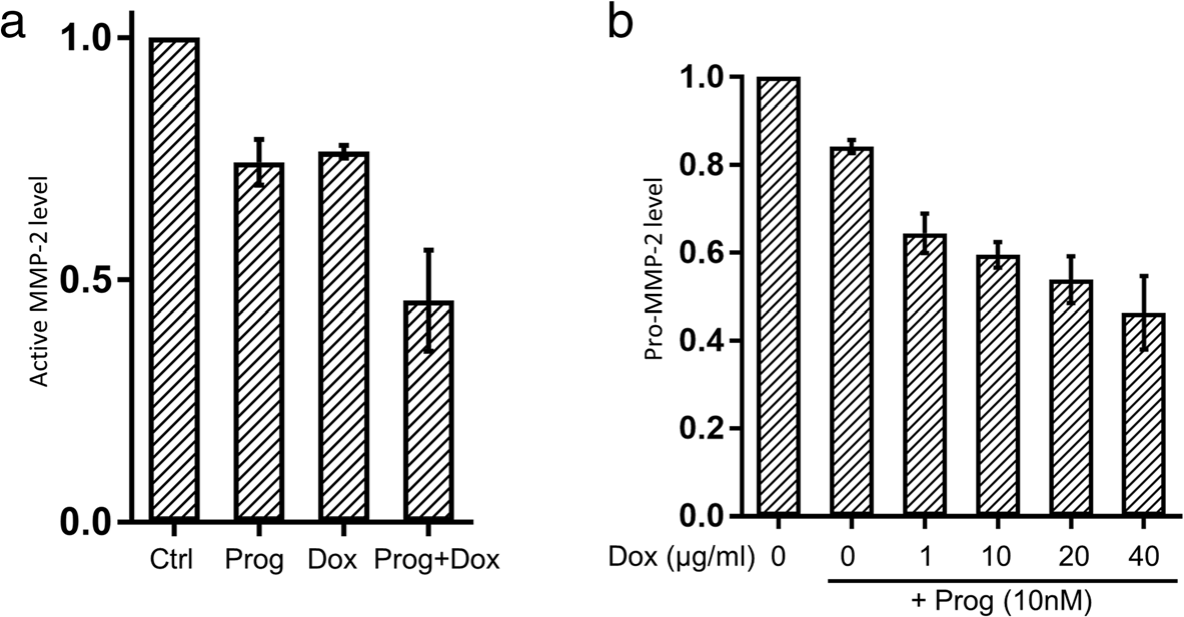
Progesterone and doxycycline have an additive effect on the reduction of active and latent MMP-2 levels. **a** 12Z endometriosis cells with either 10 nM progesterone (Prog) or 1 μg/ml doxycycline (Dox) or the simultaneous combination of both substances. The combination of low-dose doxycycline (1 μg/ml) with progesterone (10 nM) showed an additive effect on the reduction of active MMP-2. **b** Already at a low dose (1 μg/ml) doxycycline shows an additive effect to progesterone (10 nM) on inhibition of pro-MMP2. The results are represented as mean ± SD

To assess a possible synergistic effect of doxycycline with progesterone treatment, we treated 12Z with a combination of low-dose doxycycline (1 μg/ml) and progesterone (10 nM). Addition of doxycycline to progesterone further reduced levels of activated MMP-2 compared to progesterone or doxycycline alone (Fig. 4a). A further reduction of pro-MMP-2 levels in progesterone treated 12Z upon simultaneous treatment with doxycycline in a range of 1–40 μg/ml was observed especially at low doxycycline doses in zymography assays (Fig. 4b).

## Discussion

Our results demonstrate that doxycycline inhibits MMP-2 activity and expression of pro-MMP-2 in the supernatant of the 12Z epithelial endometriotic cell line and of MMP-2/− 9 and pro-MMP-2/−9in the supernatant of primary stromal endometriotic cells. Furthermore, treatment with doxycycline reduced the invasion of immortalized 12Z epithelial endometriotic cells through matrigel membranes, which served as a model for the basilar membrane of the peritoneum. The combination of doxycycline and progesterone showed synergistical effects in the reduction of MMP-2 expression in 12Z epithelial endometriotic cells.

Increased MMP-2 and -9 activity is an important characteristic of endometriotic cell infiltration as has been demonstrated in several studies [5, 14, 21–23]. It has been shown in other diseases that subantimicrobial-dose doxycycline is a potent MMP-2 and -9 inhibitor and also decreases expression of pro-MMP-2 and -9 [16, 17]. The expected range of doxycycline in human plasma has been reported at 1–3 μg/ml in pharmacokinetic studies in adults with cystic fibrosis who received doxycycline administered as an MMP inhibitor, a concentration which corresponds to the dose range used in our in vitro experiments [24]. We demonstrate in vitro, that a combination of progesterone treatment with doxycycline can considerably increase the efficacy of progesterone with regards to inhibition of invasion of endometriotic cells. To our knowledge, this is the first study proposing doxycycline in combination with a progestogen as a treatment for endometriosis.

However, a known limitation in endometriosis research is that there are very few available cell line models which only reproduce the epithelial or stromal cell component of endometriosis at once and may not behave exactly like endometriosis in vivo. This has also to be considered as a limitation of the present study since the experiments were principally done in the SV-40 transformed 12Z endometriotic cell line which is widely used in preclinical endometriosis research but may not reflect all properties of endometriotic cells in vivo. Therefore, further studies should try to investigate the effect of doxycycline in combination with a progestin in endometriotic models combining the epithelial and stromal compartment and therefore assessing its interactions or in vivo in animal models. Another limitation is the fact that MMPs are dependent on cyclic hormonal variations in vivo and that also other factors such as growth factors, cytokines, and metal ions are not likely to be present in exactly the same amount in cell culture media as compared to human peritoneal fluid [9, 14].

Endometriosis is a chronic disease with frequent recurrence after surgical treatment. Although current hormonal treatment options show good efficacy as recurrence prophylaxis, a significant subset – especially of severe forms of endometriosis – relapse under hormonal treatment [4]. Combination of hormonal and non-hormonal therapeutics may be an interesting option to enhance the success rate of current medical endometriosis treatment. Furthermore, a non-hormonal medical alternative is a frequently manifested wish of patients with endometriosis in the clinical practice. Patient preferences for an effective and well tolerated therapeutic approach with less metabolic effects are essential in the development of new drugs for endometriosis [25].

In mouse models, doxycycline reduced the size of endometriotic implants and immunohistochemical expression of MMP-2 and MMP-9 in the peritoneal cavity of mice [26]. Similar results were reported in a second in vivo study using an experimental rat model [27]. Finally, a third study in which a nanoparticle-assisted therapeutic approach was used, showed very promising results for doxycycline in the treatment of endometriosis [28]. These studies, together with our in vitro data, indicate a potential use of doxycycline as a therapeutic option in endometriosis by direct inhibition of the gelatinase activity and reduction of the infiltrative potential in endometriosis. However, clinical studies will be necessary to further evaluate the potential use of doxycycline in the treatment of endometriosis, also since there is no known ideal non-primate animal model available to adequately reflect the pathogenesis of human endometriosis.

To this date, no other studies except of the above mentioned have investigated the effects of doxycycline therapy in endometriosis with exception of clinical studies in which tetracyclines (or ethanol) were injected into endometriotic cysts at high local concentrations for sclerotherapy [29–31]. It is likely that the effect of this treatment may be related to cell damage caused by extremely high local concentrations of doxycycline and this treatment option appears obsolete nowadays. Nevertheless, it demonstrates the potential use of doxycycline in the treatment of endometriosis. It has been demonstrated that progestins reduce the expression and activity of MMP-2 and MMP-9 in human endometrial explants and the inhibitory effect of progesterone on the gelatinases MMP-2/− 9 has been proposed to be mediated by the inhibition of plasminogen activators [19]. Therefore, the additive effect of progesterone and doxycycline observed in this study may be mediated through distinct and complementary mechanisms that synergistically inhibit the expression and activity of MMP-2.

The feasibility and good tolerability of long-term subantimicrobial-dose doxycycline therapy has been demonstrated in the treatment of other diseases such as periodontosis, acne and rosacea. There were no reports of problems with bacterial resistances in the dose level used, and it is assumed that doxycycline does not exhibit antibiotic action in the low (i.e. subantimicrobial) doses used for the purpose of inhibiting the gelatinases, such as subantimicrobial-dose doxycycline (Periostat® 20 mg) used in periodontitis [32–35]. Severe allergic reactions are reportedly to be extremely rare. Doxycycline hyclate 20 mg is contraindicated in nursing mothers, pregnant women and pediatric patients due to potential teratogenic effects and potential severe adverse effects in children. Most of the adverse reactions in adults, including headache, common cold and flu symptoms, nausea, dyspepsia, joint pain and diarrhea, were reported in similar frequency for doxycycline hyclate 20 mg and for placebo [36].

From a clinical point of view a combination treatment of doxycycline with a suitable progestin, such as e.g. dienogest, for patients with severe forms of infiltrative endometriosis that do not respond well enough to the hormonal treatment alone, would be an interesting option. This treatment option could possibly be used as a medical prevention of a recurrence after surgical excision of endometriosis. Although dienogest is assumed to inhibit ovulation, an additional contraceptive barrier method would be necessary for the duration of this treatment, as is currently recommended for the therapy with dienogest alone.

## Conclusions

In conclusion, our results demonstrate the potential for use of subantimicrobial-dose doxycycline in the treatment of endometriosis in vitro and its synergistic action with progestins. Since subantimicrobial-dose doxycycline is a well tolerated drug, it appears to be a potential treatment option in endometriosis which may be further evaluated in clinical research. The combination of doxycycline with current hormonal treatment forms may increase the efficacy of hormonal treatment in endometriosis, which might be of interest in e.g. severe forms of deep-infiltrating endometriosis which are responding insufficiently to current treatment options. In addition, subantimicrobial-dose doxycycline might be an interesting alternative for patients who wish to have a non-hormonal medical prevention of recurrence after surgical excision of endometriosis.
